# A Comparative Study of Intramedullary Nail Strengthened with Auxiliary Locking Plate or Steel Wire in the Treatment of Unstable Trochanteric Fracture of Femur

**DOI:** 10.1111/os.12595

**Published:** 2019-12-21

**Authors:** Zheng‐hao Wang, Kai‐nan Li, Hai Lan, Xiao‐dong Wang

**Affiliations:** ^1^ Department of Orthopaedic Surgery Affiliated Hospital of Chengdu University Chengdu China

**Keywords:** Cerclage wire, Femoral trochanteric fracture, Locking plate, Proximal femoral intramedullary nail

## Abstract

**Objective:**

To compare the clinical outcomes of unstable femoral trochanteric fracture treated by proximal femoral intramedullary nail enhanced with lateral locking plate versus cerclage steel wire.

**Methods:**

A retrospective study was conducted on 83 patients who received open reduction and internal fixation with proximal femoral intramedullary nail combined with lateral enhanced fixations for unstable femoral trochanteric fractures from March 2015 to January 2017 in our hospital. Of these patients, 39 received the lateral enhanced fixation with locking plate, while the remaining 44 had cerclage wire as additional fixation. The clinical data were compared between the two groups.

**Results:**

All the patients in the study had surgical procedures performed smoothly. Although the plate group had significantly longer operation times and significantly higher hospitalization expenses than the wire group (*P* < 0.05), no statistically significant differences in intraoperative blood loss and hospital stay were proved between the groups (*P* > 0.05). The follow‐up period lasted for 12–24 months with an average of (16.81 ± 2.92) months. The plate group returned to full‐weight bearing significantly earlier than the wire group (*P* < 0.05). The Harris Hip Score (HHS) significantly increased in both groups over time postoperatively (*P* < 0.05). The plate group achieved a higher HHS than the wire group, which was statistically significant at 3 months (*P* < 0.05), whereas it became insignificant at 6 and 12 months postoperatively (*P* > 0.05). Regarding radiographic assessment, an excellent rate of fracture reduction was proved in 71.79% of the plate group, compared to 45.45% of the wire group, which was statistically significant (*P* < 0.05). In addition, fracture healing was achieved significantly earlier in the plate group than the wire group (*P* < 0.05); nevertheless, no statistically significant difference was noted in neck‐shaft angle at the latest follow‐up between the two groups (*P* > 0.05). At the latest follow‐up, two cases of implant loosening and two cases of coxa varus were found in the plate group, while one case of femoral head necrosis and three cases of coxa varus were revealed by radiographs in the wire group.

**Conclusion:**

The cerclage wire has benefits of saving time and operation costs; however, the locking plate has the advantages of improving fracture reduction quality, shortening time to full weight bearing and fracture healing, and improving hip function recovery due to the lateral additional fixations to proximal femoral intramedullary nail for unstable trochanteric fractures.

## Introduction

Hip fracture is considered to be the main cause of disability in the elderly in the world. By 2050, the number of hip fractures worldwide is estimated to exceed 6.3 mn per year, accounting for about half of all hip fractures 1. Unstable femoral trochanteric fracture with side wall injury will shorten the biomechanically unstable head and neck fracture broken end, which can lead to the failure of internal fixation, and increase the chance of reoperation. Factors affecting fixation stability include the geometric shape of fracture, degree of osteoporosis and comminution, selection of internal fixation, and surgical technique. The stability of rotor fracture mainly depends on the integrity of lateral wall and posterior inner wall, and the injury of greater trochanter and subtrochanteric^2^.

The choice of internal fixation will also determine the postoperative stability of unstable trochanteric fracture. The most commonly used fixations in clinic include dynamic hip screw, femoral trochanter stabilization plate, percutaneous compression plate, proximal femoral locking plate, intramedullary nail (IMN), and artificial joint replacement. IMN fixation has been recommended for the treatment of unstable trochanter fractures with good fixation stability, small intraoperative soft tissue injury, and few complications^3^. Dynamic hip screw (DHS) is not the first choice for unstable trochanteric fracture, and intramedullary fixation can be used as the first choice for patients with normal bone mass. For unstable fractures with severe osteoporosis, artificial hip arthroplasty is a safe and effective surgical choice. The unique complications for artificial hip replacement as a prosthesis implantation surgery are periprosthetic fracture, thromboembolism, prosthesis loosening, hip joint dislocation, and prosthesis wear, so it is necessary to understand the specific surgical processes associated with this procedure. For young patients, there is the problem of the useful life of an artificial joint after operation. In recent years, joint prosthesis technology and joint replacement technology are becoming increasingly mature. More and more elderly patients with unstable trochanteric fracture are treated with artificial hip replacement. However, there has been no final conclusion as to whether the primary operation, the total hip or the semi‐hip has been debated. At present, there is still no consensus on the best treatment for unstable intertrochanteric fractures. Elderly patients with severe osteoporosis may have nonunion and bone loss. Therefore, in formulating the surgical plan, orthopaedic surgeons not only need to consider the patient's physical condition, fracture type, and economic situation, but also take into account the biomechanical characteristics of the implant, and consider the stability of the lateral wall when making a reasonable choice of surgical methods.

There are still challenges in the reconstruction of lateral wall and posterior medial wall after IMN fixation of unstable trochanteric fractures. Most fractures can be reduced satisfactorily by adjusting the position of the distal limb after traction, but for the fractures of the medial and lateral wall, it is necessary to make a corresponding preoperative plan according to the type of fracture. The fracture block of the lesser trochanter of femur is easy to be pulled to the proximal end by the iliopsoas muscle, and can be corrected by adjusting the abduction of the hip joint with the traction bed. For displaced medial wall fractures, small incisions can be assisted and fixed by minimally invasive wire strapping. The fracture block of proximal trochanter of femur is easy to be pulled by gluteus medius muscle, so it is difficult to reduce it. Coarse Kirschner wire can be used to adjust the intertrochanteric fracture of the proximal femur. Repeated needle placement should be avoided to prevent complications such as splitting of the greater trochanter of the femur. The success rate of single lateral wall fixation methods, such as rotor stabilization plate and proximal femoral locking plate, is limited 4. IMN has been accepted as a way to treat unstable trochanteric fracture. It has good fixation stability, less soft tissue injury and less complications during operation. In addition, the use of encircling steel wire and tension screw to assist the fixation of intramedullary nail and lateral wall reconstruction in the treatment of unstable trochanteric fracture can reduce operative complications, shorten operation time, and result in less blood loss and less soft tissue injury. For unstable trochanteric fractures, the posterior medial wall or lateral wall can be stabilized by encircling steel wire, tension screw, and locking plate of proximal femur on the basis of IMN fixation^5^.

The choice of auxiliary fixation mode is diverse, and, at present, the choice of IMN fixation auxiliary fixation mode is still controversial in China and abroad. The purpose of this study was: (i) to explore the advantages and disadvantages of assisted anatomical locking plate for unstable femoral trochanteric fracture; (ii) to explore the advantages and disadvantages of assisted wire fixation for unstable femoral trochanteric fractures; (iii) to compare two assisted fixed methods; and (iv) to explore the timing of selection.

## Data and Methods

### *Inclusion and Exclusion Criteria*

Inclusion criteria for this study: (i) unstable trochanteric fracture: 31A2.2, 31A2.3 and 31A3 intertrochanteric fracture and subtrochanteric fracture^6^; (ii) age > 18 years old; (iii) unilateral primary closed trochanteric fracture; (iv) the auxiliary steel plate or encircling steel wire was fixed with intramedullary nail; (v) the follow‐up period was more than 1 year.

Exclusion criteria for this study: (i) Patients who could not walk before fracture and other fractures affected rehabilitation; (ii) patients with old fracture; (iii) patients with compound hip trauma and pathological fracture; (iv) patients with incomplete case data and follow‐up time of less than 1 year.

### *General Information*

From March 2015 to January 2017, 83 patient cases met the inclusion criteria; of these, there were 41 males and 42 females. The age range was 46 to 89 years, with an average of (69.27 ± 11.92) years. According to the different methods of auxiliary fixation, the patients were divided into steel plate group (39 cases) and steel wire group (44 cases). The general data of the two groups were shown in Table 1. There was no significant difference in age, sex, injury mechanism, and osteoporosis between the two groups. AO classification was used for intertrochanteric fracture and Seinsheimer classification for subtrochanteric fracture.

**Table 1:** Comparison of general data between two groups of different auxiliary fixation methods

Indicators	Steel Plate group(39 cases)	Steel wire group (44 cases)	Statistical value	*P* value
Age (years, mean ± SD)	69.92 ± 11.34	68.68 ± 12.52	*t* = 0.471	0.639
Gender (cases, male / female)	20/19	21/23	χ^2^ = 0.105	0.827
Cause of injury (cases, high fall/ accident / fall)	7/17/15	8/15/21	χ^2^ = 0.894	0.679
Osteoporosis(cases, yes / no)	22/17	23/21	χ^2^ = 0.143	0.826
Fracture type (Intertrochanteric / Subtrochanteric fracture)	23/16	30/14	χ^2^ = 0.759	0.493
Intertrochanteric(AO31A2.2/A2.3/A3.1/A3.2/A3.3)	3/5/7/8/5 (28)	4/6/7/5/3(25)	χ^2^ = 1.391	0.889
Subtrochanteric fracture (Seinsheimer I/II/III/IV)	0/2/3/6 (11)	0/6/9/4 (19)	χ^2^ = 3.265	0.225

### *Surgical Method*

The patient took the supine position for traction reduction. The broken end of the fracture and traction reduction was determined by C‐arm fluoroscopy. A 5 cm longitudinal slit was made above the greater trochanteric to expose the greater trochanteric, and the needle point was determined at the inside 1/3. The guide needle was inserted along the longitudinal axis of the Patient's body. C‐arm fluoroscopy confirmed that the guide needle was in the correct position. Reaming in the direction of the guide needle, the intramedullary nail was rotated and installed. A deflector was used to insert the guide needle into the femoral head and examine the position by fluoroscopy. The spiral blade was placed after measuring the depth. The screw was placed in the center of the femoral head or slightly below the sub‐cartilage edge of the lower 0.5–1 cm, and then place the distal locking screw on the distal femur.

In the steel plate group, without prolonging the incision, the lateral thigh muscle was peeled off to the distal end of the femur, and the locking plate was placed in the lateral wall through this space. The position of the plate was identified by fluoroscopy, and the position of the distal locking hole was marked at the same time. The intramedullary nail was avoided, and the locking nail was placed into the internal and proximal femoral shaft of the femoral neck. The distal femur used a well‐located percutaneous, minimally invasive incision to explore the locking hole, place the locking sleeve, and place the locking nail.

In the steel wire group, through the original incision at the level of the lesser trochanter, the two parts of the steel wire banding device were connected in the front and rear of the femur and passed through the encircling steel wire. The steel wire banding device was removed, the steel wire position was adjusted up and down and tightened, and the hip joint was moved to ensure the bone mass of the lateral wall and posterior medial wall was still stable. The number of encircling steel wires were increased if necessary, and the steel wire was strengthened at the proximal end of the intramedullary nail. X‐ray confirmed whether the reset was satisfactory.

### *Evaluation Index*

The amount of blood loss (according to Gross formula^7^) and operation time were recorded for the two groups. The occurrence, hospitalization time, and cost of operation‐related complications were recorded. The function of hip joint was evaluated by Harris Hip Score (HHS), and the time of complete weight bearing was recorded. The quality of fracture reduction was evaluated by modified Baumgaertner standard^6^. The fracture healing time was recorded^7^, and the collodiaphyseal angle was measured.

#### *Blood Loss*

Gross formula: For assessing Actual blood loss (ABL) in surgery. **ABL** = **BV**×[**Hct**_i_‐**Hct**_f_]/**Hct**_m_. Total Body volume (BV, Body volume) = 70 ml × Body weight (kg). **Hct**_i_: Before surgery, **Hct**_f:_ End of surgery. **Hct**_m_: (**Hct**_i_ + **Hct**_f_) /2.

#### *Baumgaertner Standard*

To evaluate the quality of fracture reduction, the following two items are excellent and one is good. If neither of the following is in line with each other, it is poor. The results showed: (i) in the positive position of X‐ray, the collodiaphyseal angle was normal or slightly valgus, and the lateral angle was less than 20°; and (ii) the reduction was completed in the positive and lateral position of the main fracture, and the shortening was less than 0.5 cm^8^.

#### *Fracture Healing Standard*

There was no tapping pain in the hip, no longitudinal percussion pain, no local pain. At the same time, X‐ray or CT indicates broken end connection of fracture, and complete bony callus formation is regarded as fracture healing^9^_._

#### *Harris Hip Score (HHS)*

The HHS was used to evaluate postoperative recovery of hip function in an adult population. The HHS score system mainly includes four aspects: pain, function, absence of deformity, and range of motion. The score standard had a maximum of 100 points (best possible outcome). A total score < 70 is considered as poor score, 70–80 as fair, 80–90 as good, and 90–100 as excellent.

#### *Collodiaphyseal Angle*

The angle between the longitudinal axis of the femoral shaft and the axis of the femoral neck is the collodiaphyseal angle. The normal range of collodiaphyseal angle was between 110°–140°, with an average of 128°, 132° in male and 127° in female. If this angle is less than the normal range, it is hip varus, and if it is greater than the normal range, it is hip valgus.

### *Statistical Method*

SPSS22.0 software was used for statistical analysis. The counting data were tested by Chi‐square test. The measurement data obeying normal distribution and homogeneous variance were described as (mean ± SD). The *t*‐test of independent samples was used between the two groups. Does not obey the normal distribution or the variance is uneven, uses the rank sum test. One‐way ANOVA was used for intra‐group comparison. The test level *α* was 0.05 on both sides.

## Results

### *Perioperative Situation*

The operation was completed successfully in both groups. In the intraoperative plate group, the fat on the lateral thigh of three patients was too thick, and the modified minimally invasive percutaneous plate osteosynthesis (MIPPO) minimally invasive placement of the lateral locking plate was difficult, so the plate was placed under direct vision after prolonging the incision. In the steel wire group, 12 patients with severe fracture displacement of medial and lateral wall injury were treated with prolonged incision and multi‐strand steel wire encircling and strengthening during the operation. The perioperative data of the two groups are shown in Table 2. The operation time in the steel plate group was significantly longer than that in the steel wire group (*P* < 0.05), but there was no significant difference in average blood loss and average hospital stay between the two groups (*P* > 0.05). The average hospitalization cost in the steel plate group was significantly higher than that in the steel wire group (*P* < 0.05). In the steel plate group, postoperative swelling was obvious in three cases, and symptoms were relieved after ice compression and elevation of the affected limbs. The gastrointestinal reactions were relieved after symptomatic treatment in three cases. In the steel wire group, one case had subcutaneous fluid, which was bandaged under pressure, and the wound was stable after dressing change. Four cases of gastrointestinal reaction in this group were relieved after symptomatic treatment. There was no wound infection and no cardiovascular and thrombus complications in both groups.

**Table 2:** Peri‐operative data (mean ± SD) and comparison between the two groups

Indicators	Steel Plate group (39 cases)	Steel wire group (44 cases)	Statistical value	*P* value
Operation time (min)	79.05 ± 11.84	69.57 ± 12.34	*t* = 3.561	0.001
Blood loss (mL)	247.31 ± 20.60	244.95 ± 20.49	*t* = 0.521	0.604
Days of hospitalization	14.64 ± 3.38	15.13 ± 3.32	*Z* = ‐0.867	0.386
Hospitalization expenses (10,000 RMB)	3.68 ± 0.35	3.13 ± 0.44	*Z* = ‐5.033	<0.001

### *Follow‐up Results*

Eighty‐three patients were followed up for 12–24 months, with an average of (16.81 ± 2.92) months. During the follow‐up, one patient in the steel wire group underwent total hip replacement because of osteonecrosis of the femoral head and severe hip pain 19 months after operation. The follow‐up measurement data of the two groups are shown in Table 3. The average time of complete weight loading in the steel plate group was significantly earlier than that in the steel wire group, the HHS in the two groups increased significantly with the prolongation of time, and there was significant difference at different time points (*P* < 0.05). Three months after operation, the HHS in the steel plate group was significantly higher than that in the steel wire group (*P* < 0.05). At 6 and 12 months after operation, the HHS in the steel plate group was still higher than that in the steel wire group, but the difference was not statistically significant (*P* > 0.05, Table 3).

**Table 3:** Follow‐up measurement data (mean ± SD) and comparison between the two groups

Indicators	Postoperative time point	Steel Plate group (39 cases)	Steel wire group (44 cases)	*t* value	*P* value
Full weight bearing time (days)		84.49 ± 10.74	90.66 ± 8.47	−2.922	0.005
Harris score	3 months	75.46 ± 6.99	71.48 ± 8.47	2.320	0.023
	6 months	81.13 ± 7.41	80.23 ± 6.76	0.579	0.564
	12 months	87.92 ± 6.91	86.16 ± 5.43	1.300	0.197
	*F* value	30.033	49.065		
	*P* value	<0.001	<0.001		

During the last follow‐up, of the 39 cases in the steel plate group, 18 were completely painless, 19 had mild pain, two had obvious pain, 25 walked normally, 11 had mild claudication, two had obvious claudication, one walked with crutches, 24 had normal squatting activity, 13 had mild squatting limitation, two had obvious limited squatting activity, 23 recovered their pre‐injury exercise and labor ability, and 16 cases did not return to the level of pre‐injury exercise and labor ability. Of the 44 cases in the steel wire group, 19 were completely painless, 22 had mild pain, three had obvious pain; 26 were walking normally, (no claudication), 14 had mild claudication, two had claudication; 25 had normal squatting ability, 17 had mild squatting limitation, two had obvious squatting limitation, 28 had recovery of pre‐injury exercise and labor ability, and 16 did not recover to the level of pre‐injury exercise ability.

### *Image Evaluation*

The imaging evaluation data of the two groups are shown in Table 4. According to the modified Baumgaertner standard, the excellent rate of fracture reduction quality was 71.79% in the steel plate group and 45.45% in the steel wire group. There was significant difference between the two groups (*P* < 0.05). The fracture healing time in the steel plate group was significantly earlier than that in the steel wire group (*P* < 0.05). At the last follow‐up, there was no significant difference in collodiaphyseal angle between the two groups (*P* > 0.05).

**Table 4:** Comparison of imaging evaluation results between two groups

Indicators	Steel Plate group (39 cases)	Steel wire group (44 cases)	Statistical value	*P* value
Reduction of fracture (cases, excellent / good / fair)	28/10/1	20/22/2	*χ* ^2^ = 5.917	0.034
Fracture healing time (mean ± SD, days)	93.46 ± 7.87	100.02 ± 8.89	*t* = −3.539	0.001
Last follow‐up collodiaphyseal angle (°)	128.18 ± 11.07	127.25 ± 12.16	*t* = 0.362	0.718

**Figure 1:**
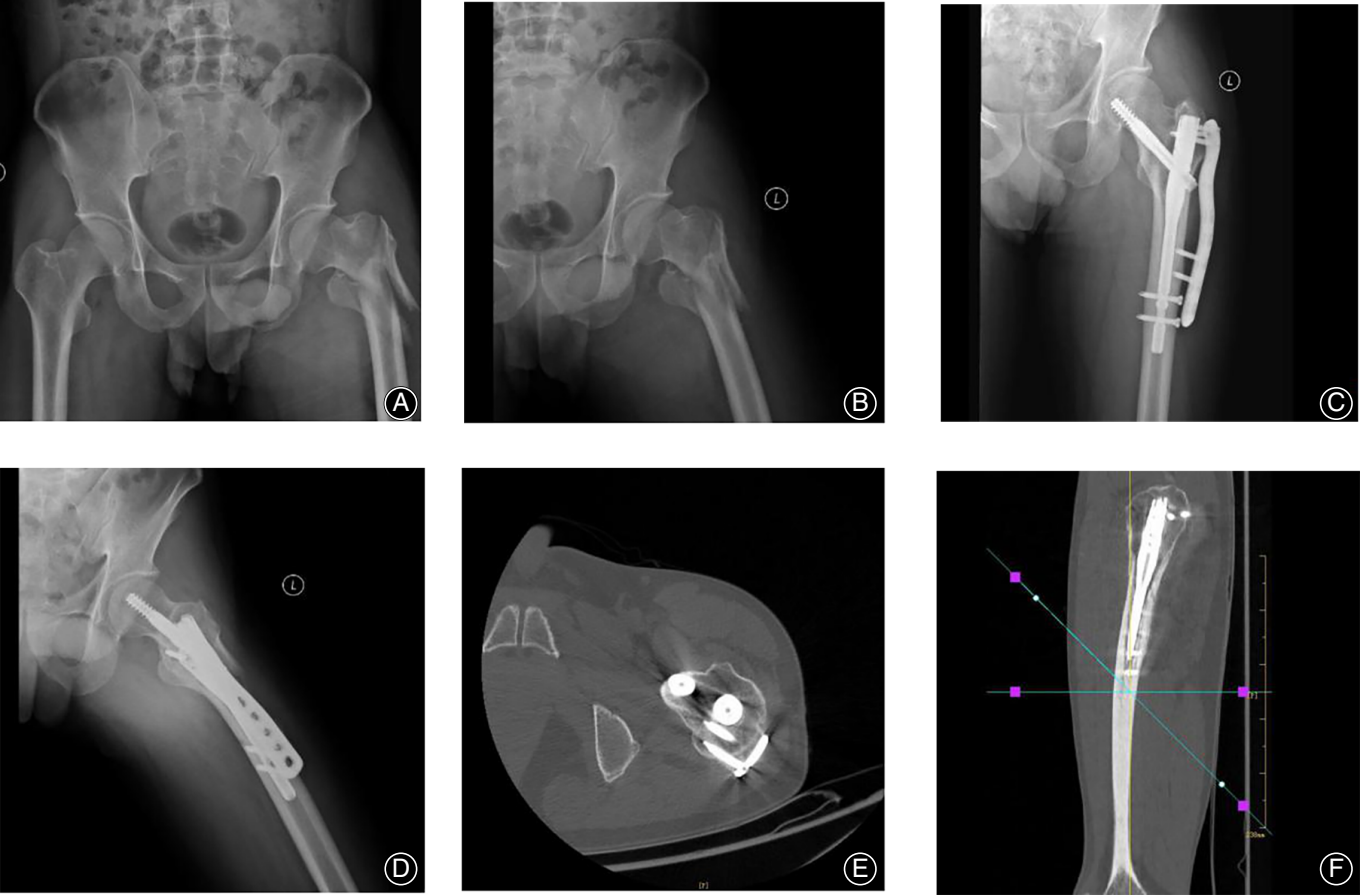
A 57‐year‐old male patient with trochanteric fracture was treated with intramedullary nail assisted anatomical locking plate fixation of the lateral wall of the proximal femur. (A, B): X‐ray lateral wall injury was serious before operation. (C, D): After operation, X‐ray positive and lateral films showed anatomical reduction and reliable fixation of fracture. (E, F): 6 months after operation, CT showed bone healing.

**Figure 2:**
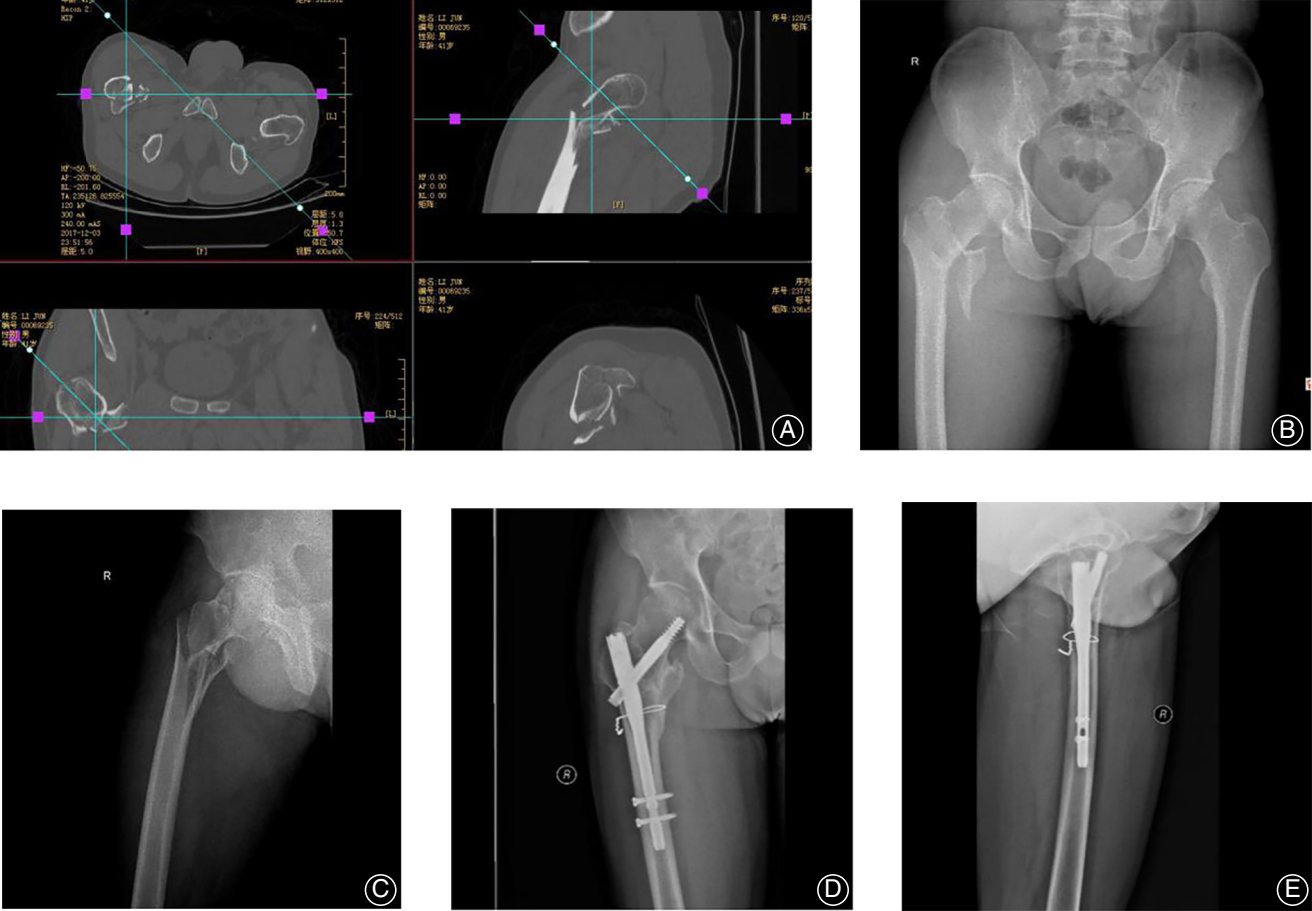
A 41‐year‐old male patient with trochanteric fracture was treated with intramedullary nail fixation and steel wire encircling fixation. (A): preoperative CT and reconstruction showed obvious fracture displacement, (B, C): preoperative X‐ray positive and lateral position showed serious lateral wall injury. (D, E): After operation, X‐ray positive and lateral position showed anatomical reduction and good fixation of fracture.

At the last follow‐up there was no spiral blade cutting the femoral head in the locking plate group, one case of posterior medial wall fracture line was still visible, but the trochanteric fracture node had continuous callus, and two cases of plate and screw unnailed, but the fracture healed completely. Hip varus was found in two cases and the fracture healed completely. There was no spiral blade cutting the femoral head in the steel wire group, two cases of lateral wall fracture nonunion, but the posterior medial wall and trochanteric fracture healed completely, one case of osteonecrosis of the femoral head and three cases of hip varus. The images of typical cases are in Figs 1 and 2. In eight patients with 39 cases of plate fixation, the anatomical locking plate was removed at the early stage, and then the intramedullary nail was removed after the second operation.

## Discussion

There are still many disputes about the choice of internal fixation and surgical scheme for femoral trochanteric fracture, especially unstable fracture 10, 11. Femoral shaft displacement and varus collapse are common complications of unstable trochanteric fracture. A number of studies have shown that the integrity of the lateral wall is a key factor in hip varus^12^. Compared with dynamic hip screw, intramedullary nail fixation has better stability, and reduces the incidence of fracture varus and collapse^13^, but there may be injury or aggravation of lateral wall injury when intramedullary nail is inserted. There are challenges in the operation of lateral wall fractures associated with unstable fractures of the trochanteric. Three‐dimensional reconstruction CT scan showed that most of the greater trochanteric fractures occurred in the coronal plane, and were serious enough to lead to a high degree of instability. Therefore, the sidewall of the greater rotor is recommended for reconstruction to provide stability. In many cases, the intramedullary fixation passes through the middle of the greater trochanteric segment, and the stability of the lateral wall cannot be guaranteed without the auxiliary fixation of the lateral wall. Circumferential steel wire and proximal femoral locking plate can be used to fix greater trochanter fracture on coronal plane. The proximal femoral locking plate and steel wire were used to realize the anatomical reduction of the fracture and to provide a stable lateral wall. The surgical approach to stabilize the medial wall is difficult.

For the posterior medial wall collapsed fractures, it is difficult to reconstruct them with either cerclage wire or locking plate. It poses an even greater challenge when the patients have osteoporotic bone. The lesser trochanter and calcar femorale are the most important pressure conduction sites in the medial wall of the trochanteric, and the integrity of posterior medial wall directly affects the stability of trochanteric. The fracture block of posterior medial wall was fixed by steel wire as far as possible, and the steel wire clung to the bone cortex of posterior medial wall in this process. During the operation, it is necessary to make sure that the encircling of the steel wire does not affect the activity of the adductor muscle. The advantage of auxiliary steel wire bandaging is that the operation time is short, the operation cost is relatively low, and the medial wall has the effect of encircling and fixing. The locking plate of proximal femur is helpful to shorten the time of complete weight bearing and fracture healing, and improve the early functional recovery of hip joint. The quality of fracture reduction is better than that of encircling steel wire.

Screw cutting of the femoral head is a serious complication of intramedullary nail fixation. The protruding tip of the screw causes obvious pain and disability in the hip joint, leading to the formation of osteoarthritis of the hip joint. In the past, it was considered that the cusp distance (TAD) of screws was the key factor affecting the screw cutting rate. It was suggested that the TAD standard should be greater than 15 mm to reduce screw displacement. In the case of poor angular reduction, the screw is not parallel to the femoral neck, which greatly reduces the possibility of the best nail position and the best TAD, and the anatomical fracture reduction is helpful for more suitable nail placement, and the significance of good reduction is greater than that of screw position or TAD. Some studies have shown that TAD cannot fully predict the removal rate of screws 14. Reasonable placement and adequate reduction and osteoporosis also affect screw cutting of the femoral head^15^. The comparative study of different types of intramedullary fixation shows that fracture collapse is an important factor leading to screw incision. Fogagnolo *et al*.^8^ confirmed that the resection rate was as high as 10% in patients with unstable trochanteric after intramedullary fixation, emphasizing the necessity of anatomical reduction of fracture. Huang *et al*. also pointed out that intramedullary nail fixation in patients with osteoporosis is insufficient, and screw cutting penetration rate depends on the size of intramedullary nail, good reduction and lateral wall stability^16^. Posterior medial and lateral wall reduction and fixation will reduce the risk of screw removal and reduce complications associated with fixation failure and reoperation. The resection rate was associated with poor reduction of femoral collodiaphyseal angle after fixation, but the most closely related risk factor was lateral wall fracture^17^, ^18^. All these studies emphasize the importance of reduction quality after intramedullary fixation of unstable trochanteric fractures. For unstable fracture of trochanteric, the reduction quality of intramedullary nail fixation with steel plate is better than that of steel wire encircling fixation.

Auxiliary fixation is particularly important for medial and lateral wall reduction and fixation after intramedullary nail fixation. For the repair of lateral wall stability, proximal femoral locking plate is better than steel wire encircling, and the complete fracture healing time is shorter. In this study, there was no significant difference in operation‐related complications between the two groups, and there was no difference in the recovery of hip joint function between the two groups, 6 and 12 months after operation. As for the elderly patients who do not require high hip joint activity, they can choose steel wire encircling fixation. This study confirmed that the fracture healing time and complete weight‐bearing time of steel wire encircling fixation was longer than that of proximal locking plate. In patients with severe osteoporosis, the incidence of recurrent fracture of trochanteric fracture within 3 years after operation is about 2.4%. It is suggested that patients have stable fixation during operation, early functional exercise, and regular anti‐osteoporosis treatment^19^, ^20^. Long‐term bed rest will aggravate bone mass loss, aggravate osteoporosis, and increase the risk of complications in bed rest. For patients with severe osteoporosis, it is recommended to use proximal femoral locking plate fixation for the treatment of intertrochanteric fracture. Early weight‐bearing activities of the hip joint and strengthen the functional exercise of the hip joint. However, for patients with severe osteoporosis, the anatomical reconstruction plate of the lateral wall is not recommended. For the patients with collapsed fractures in the lateral wall or a very thin lateral wall, it was difficult to reconstruct the lateral wall by using a locking plate, especially as the locking screws can likely only be fixed unicortically. The bone condition will affect the stability of the steel plate. If the osteoporosis is serious and the fixation of the steel plate is not firm, it will lose its significance. Multiple strands of steel wire can be used to fix the lateral wall. For patients with severe medial wall injury, it is recommended to combine steel wire encircling on the basis of locking plate at the proximal end of the femur. It is suggested that the appropriate auxiliary fixation should be selected by comprehensively considering the type of fracture, lateral wall injury, age, basic diseases, osteoporosis, functional requirements of hip joint, economic situation, and so on.

The advantage of this study is that the two sets of general data are compared. For type A2, steel wire encircling is selected because the fracture is relatively not serious, and the proximal locking plate is selected for serious rotor injury of type A3, both of which will affect the experimental results. Through the comparison of AO typing between the two groups, this influencing factor was excluded. The possibility of different severity of fracture in the two groups before surgical intervention was excluded, which led to different fracture healing after operation. At the same time, the two groups of osteoporosis were considered, and the factors affecting the prognosis of the fracture were excluded as much as possible. At the same time, this study also has shortcomings. When reviewing the surgical‐related complications, the sample size is limited, and the single complications are relatively few. The results may be inaccurate. In future studies, the sample size can be increased, the follow‐up time can be extended, and complications can be observed.
